# Axial elongation among Hong Kong myopic children and adolescents wearing single vision spectacles from a clinical setting

**DOI:** 10.1038/s41598-025-99954-1

**Published:** 2025-05-03

**Authors:** Kryshell Yu Qi Wong, Rachel Ka Man Chun, Andrew Kwok Cheung Lam

**Affiliations:** 1 Centre for Myopia Research, School of Optometry, The Hong Kong Polytechnic University, Hung Hom, Kowloon, Hong Kong; 2 Centre for Eye and Vision Research (CEVR), 17W Hong Kong Science Park, Sha Tin, Hong Kong; 3 Research Centre for SHARP Vision (RCSV), The Hong Kong Polytechnic University, Hung Hom, Kowloon, Hong Kong

**Keywords:** Epidemiology, Epidemiology, Refractive errors

## Abstract

This retrospective cohort study investigates axial elongation in myopic children and adolescents wearing single vision spectacles, before and after the availability of proven myopia control spectacles. Electronic clinical records of patients aged 3–17 years who were prescribed single vision spectacle lenses between July 2012 and August 2013 (2012_13 cohort) and between July 2018 and August 2019 (2018_19 cohort) were analyzed. Follow-up duration ranged from 12 to 51 months, and axial elongation was normalized to a 12-month change. There were 330 and 165 patients in the 2012_13 and 2018_19 cohorts, respectively. The annualized axial elongation, expressed as mean (standard error), was 0.27 (0.01) mm for the 2012_13 cohort and 0.26 (0.01) mm for the 2018_19 cohort. In both cohorts, median annual axial growth was ≥ 0.40 mm at ages 3–5, ≥ 0.30 mm at ages 6–8, ≥ 0.20 mm at ages 9–10, ≥ 0.10 mm at ages 11–13, and < 0.10 mm at ages 14–17. Younger age was closely associated with faster annualized axial elongation, with each year of increase in baseline age results in a decrease in annualized axial growth by 0.04 mm. These findings serve as a useful reference for future myopia control trials to assess treatment efficacy.

**Supplementary Information:**

The online version contains supplementary material available at 10.1038/s41598-025-99954-1.

## Introduction

The global prevalence of myopia has been increasing for years, presenting considerable challenges to public health, and imposing a substantial economic burden on society^1^. Projections indicate that by 2050, approximately half of the world’s population will be affected by myopia^2^. Its prevalence is particularly alarming in East and Southeast Asia, surpassing 90% of schoolchildren living in specific urban areas^3^. While other parts of the world exhibit lower prevalence rates of 20–50%, they remain higher than those in past generations^4–6^.

Myopia is commonly characterized by abnormal axial elongation, in which axial length (AL) growth exceeds the reduction in refractive power of the cornea and lens^7,8^. Recently, researchers have shown a growing interest in AL owing to its rapid and non-invasive measurement and the dispensability of cycloplegia, particularly among young children and orthokeratology lens wearers^9^. Previous studies have also suggested a stronger correlation of visual impairment with AL rather than refractive error^10^, with excessive AL growth increasing the risk of retinal detachment, myopic macular degeneration, cataracts, and glaucoma^11^. Therefore, the fundamental goal in controlling myopia is to inhibit axial elongation, thereby reducing the risk of axial growth-related myopic complications^9,12^.

A recent meta-analysis reported an annual axial elongation of approximately 0.50 mm in Asians and 0.30 mm in Caucasians aged 8 years, decreasing to 0.30 mm and 0.20 mm, respectively, at 11 years^13^. Defining the expected range of axial growth rates at different ages allows clinicians to identify children at risk of excessive axial elongation and determine whether the treatment efficacy for a specific myopia control intervention is adequate. However, these axial elongation data were primarily obtained from untreated control groups in randomized controlled trials (RCTs), where, despite randomization, participants were often selected based on stringent eligibility criteria that could limit their external validity^14^. As a result, the reported axial elongation trends may not fully reflect those observed in clinical practice.

Therefore, this study aimed to investigate the axial elongation patterns among Hong Kong myopic children and adolescents wearing single vision (SV) spectacles in a clinical setting, considering factors such as age at initial presentation and sex. Given the increasing availability of myopia control interventions over the past decade, it is believed that axial growth rate among children and adolescents who wear SV spectacles nowadays may be lower compared to those who wore SV spectacles in the past, when limited myopia control options were available. Thus, this study also compares annual axial growth rates before and after the introduction of defocus incorporated multiple segments (DIMS) spectacle lenses into clinical practice, as DIMS is the first spectacle lens design proven to effectively slow down myopia progression and axial elongation^15^. The findings of this study may provide valuable insights for future clinical trials aimed at assessing the efficacy of myopia control interventions in slowing axial elongation, particularly in light of the growing ethical concerns regarding the inclusion of untreated control groups.

## Results

**Fig. 1:**
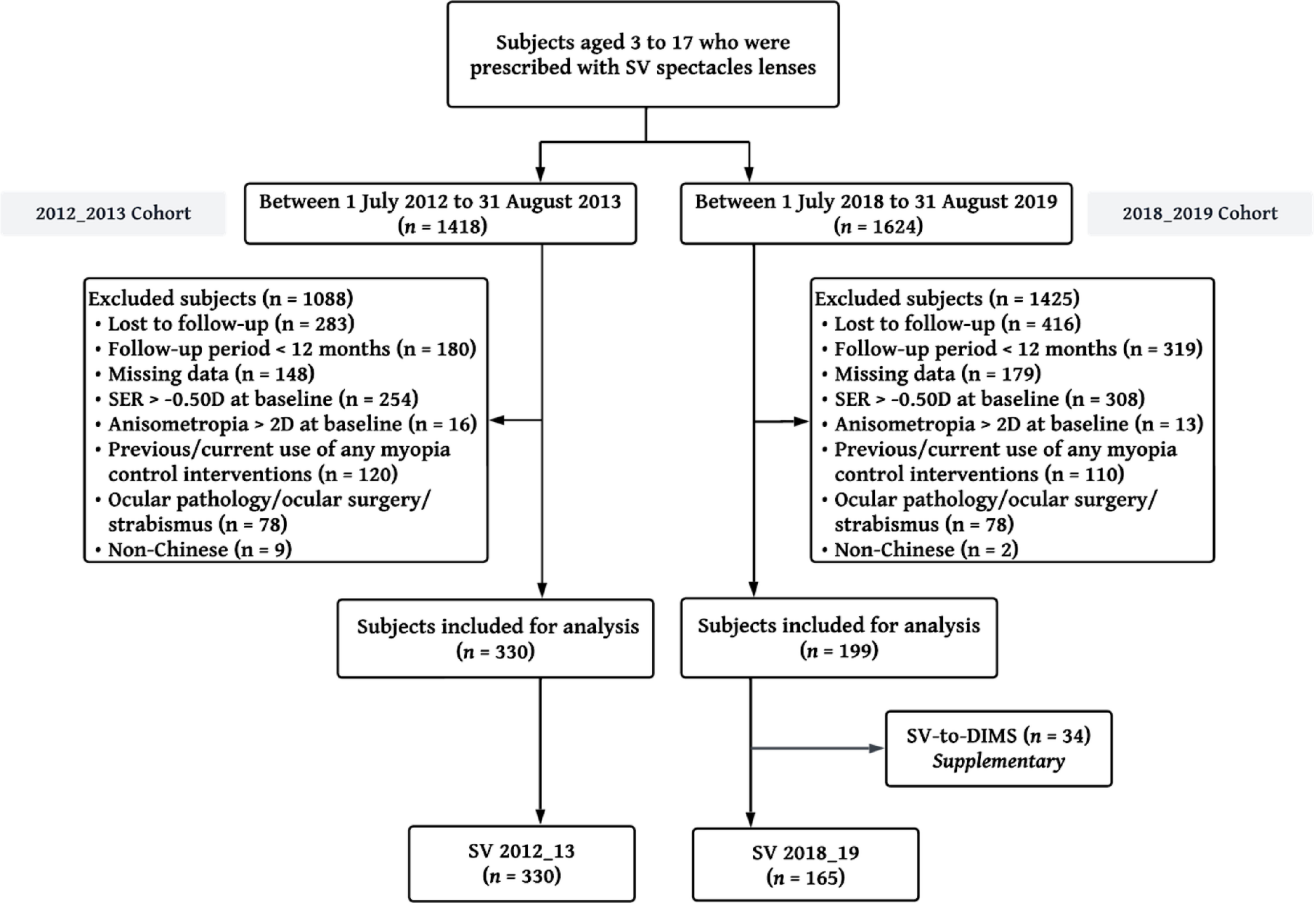
Flowchart demonstrating retrospective cohort study design. DIMS, defocus incorporated multiple segment; SV, single vision; SER, spherical equivalent refraction.

Of the 1418 and 1624 patient records reviewed for the 2012_13 and 2018_19 cohorts, 1088 and 1425 patients were excluded from the respective cohorts, most commonly because of loss to follow-up after the baseline visit, SER > − 0.50D and a follow-up period of less than 12 months (Fig. 1).

In total, 330 patients were included in the 2012_13 cohort and 165 patients in the 2018_19 cohort. Table 1 demonstrates the baseline characteristics of SV spectacles wearers in the 2012_13 and 2018_19 cohorts. At baseline, the 2012_13 cohort was significantly more myopic (median [IQR]: − 2.25 [–4.00, − 1.34] vs. − 1.63 [–3.06, − 1.00] D, *P* < .001), and had a longer AL (median [IQR]: 24.38 [23.64, 25.25] vs. 24.19 [23.35, 25.00] mm, *P* = .032) than the 2018_19 cohort. Cycloplegic refraction was conducted in 30% (2012_13 cohort) and 40% (2018_19 cohort) of patients. There were no significant differences in the percentage of male and female participants between the two cohorts (*P* = .405).

**Table 1:** Baseline characteristics of the SV 2012_13 and SV 2018_19 cohorts.

Baseline characteristics, median [IQR]	SV 2012_13 (*n* = 330)	SV 2018_19 (*n* = 165)	*P* value
Age, years	9.00 [7.00–12.00]	9.00 [6.00–11.00]	0.141
Gender			
Male, no. (%)	189 (57.30)	88 (53.30)	0.405
Female, no. (%)	141 (42.70)	77 (46.70)	
Cycloplegic refraction			
Yes, no. (%)	100 (30.30)	66 (40.00)	0.031*
No, no. (%)	230 (69.70)	99 (60.00)	–
Spherical power, D	− 2.00 [− 3.50 to − 1.00]	− 1.25 [− 2.25 to − 0.50]	< 0.001**
Cylindrical power, D	− 0.75 [− 1.75 to − 0.25]	− 0.75 [− 2.00 to − 0.25]	0.978
SER, D	− 2.25 [− 4.00 to − 1.34]	− 1.63 [− 3.06 to − 1.00]	< 0.001**
Axial length, mm^†^	24.38 [23.64 to 25.25]	24.19 [23.35 to 25.00]	0.032*
Follow-up duration, months	34.55 [24.45 to 40.40]	29.10 [23.60 to 36.10]	< 0.001**
Anisometropia, D	− 0.13 [− 0.50 to 0.25]	0.00 [− 0.31 to 0.38]	0.160

Mann–Whitney U test or ^†^unpaired t-test was used for continuous data, and Chi-Square test for categorical data, *P < .05, **P < .01. D, dioptres; IQR, interquartile range; SER, spherical equivalent refraction; SV, single vision.

The unadjusted median annualized axial elongation and myopia progression were comparable between the 2012_13 and 2018_19 cohorts (change in AL: *P* = .198; change in SER: *P* = .774; Table 2). After adjusting for covariates, the GEE model also revealed similar annualized axial elongation (*P* = .486) and myopia progression (*P* = .109) in the two cohorts. The adjusted AL changes reported as mean (SE) were 0.27 (0.01) and 0.26 (0.01) mm/year in the 2012_13 and 2018_19 cohorts, respectively. In the GEE model, age and follow-up duration (both *P* < .001) were closely associated with the annualized AL change (Table 3). When the baseline age and follow-up period increased, annualized axial elongation decreased. Similarly, annualized myopia progression was slower when the baseline age and follow-up period (both *P* < .001) increased. In contrast, less myopic SER at baseline was associated with faster annualized myopia progression (*P* = .013).

**Table 2:** Unadjusted and adjusted annualized AL and SER changes in the SV 2012_13 and SV 2018_19 cohorts.

Outcomes	SV 2012_13 (*n* = 330)	SV 2018_19 (*n* = 165)	*P* value
Unadjusted model, median [IQR]^a^			
Change in AL, mm/year	0.23 [0.10–0.38]	0.25 [0.13–0.40]	0.198
Change in SER, D/year	− 0.37 [− 0.69 to − 0.16]	− 0.38 [− 0.67 to − 0.17]	0.774
Adjusted model, mean (SE)^b^			
Change in AL, mm/year^‡^	0.27 (0.01)	0.26 (0.01)	0.486
Change in SER, D/year ^†^	− 0.47 (0.02)	− 0.41 (0.03)	0.109

^a^Mann–Whitney U test; ^b^GEE model, *P < .05. ^‡^Mean (SE) adjusted for age, follow-up duration and baseline AL as covariates. ^†^Mean (SE) adjusted for age, follow-up duration and baseline SER as covariates. AL, axial length; D, dioptres; IQR; interquartile range; SE, standard error; SER, spherical equivalent refraction; SV, single vision.

**Table 3:** GEE model adjusted for potential factors and covariates associated with annualized AL and SER changes.

Factors & covariates	Change in AL, mm/year	
	β (95% Confidence interval)	*P* value
Groups (SV2012_13/SV2018_19)	0.011 (− 0.020 to 0.041)	0.486
Gender (Male/Female)	− 0.013 (− 0.043 to 0.017)	0.406
Age, years	− 0.040 (− 0.047 to − 0.033)	< 0.001**
Baseline AL, mm	− 0.001 (− 0.016 to 0.014)	0.893
Follow-up duration, months	− 0.004 (− 0.006 to − 0.002)	< 0.001**

Factors & covariates	Change in SER, D/year	
	β (95% Confidence interval)	*P* value
Groups (SV2012_13/SV2018_19)	− 0.057 (− 0.127 to 0.013)	0.109
Gender (Male/Female)	0.009 (− 0.056 to 0.074)	0.787
Age, years	0.043 (0.029 to 0.057)	< 0.001**
Baseline SER, D	− 0.027 (− 0.048 to − 0.006)	0.013*
Follow-up duration, months	0.007 (0.003 to 0.011)	< 0.001**

Statistical significance value, *P < .05, **P < .01. AL, axial length; D, dioptres; SER, spherical equivalent refraction; SV, single vision.

**Fig. 2:**
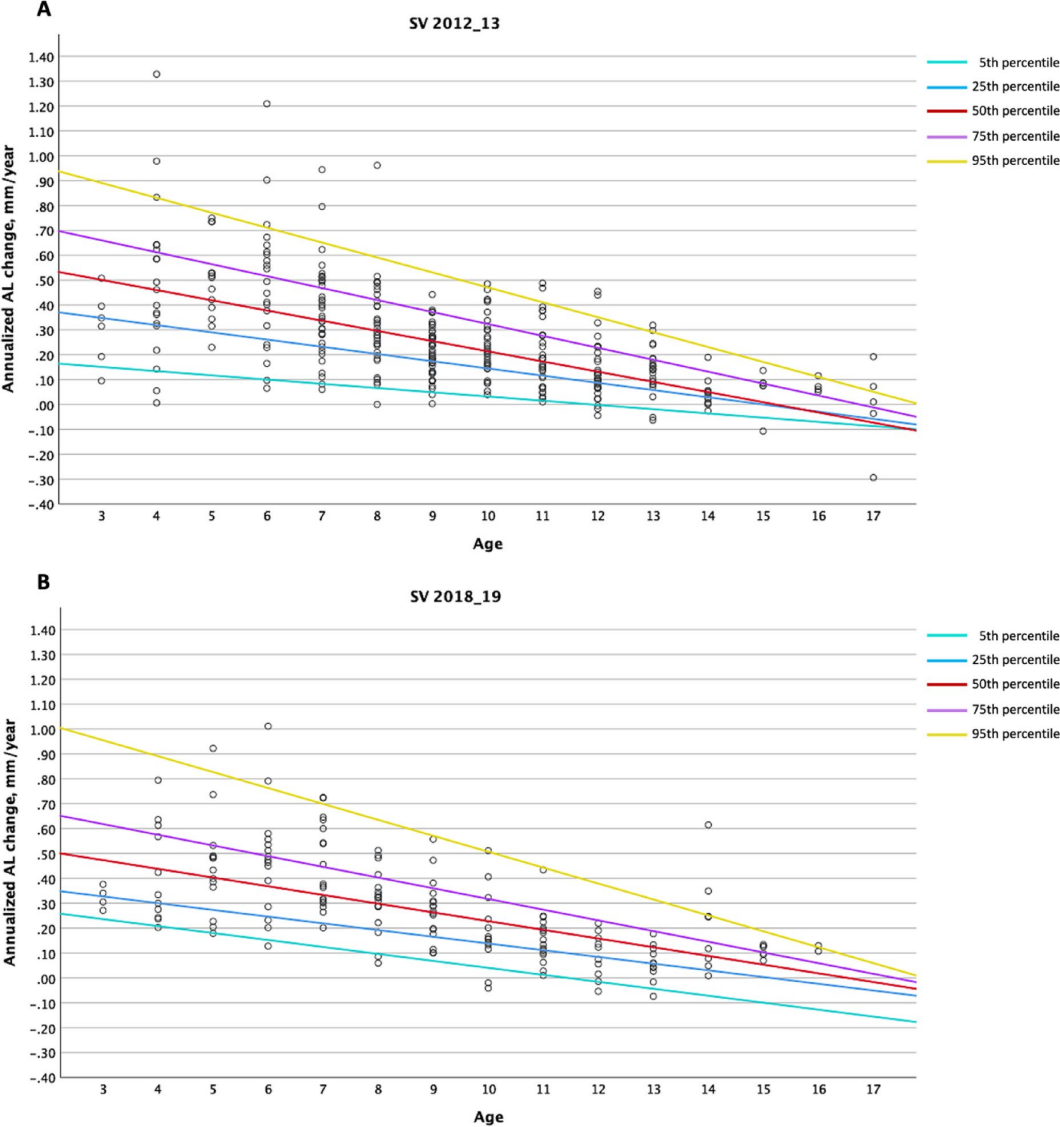
Percentile curves for age-specific annual axial growth (mm/year) in the (**A**) SV 2012_13 and (**B**) SV 2018_19 cohorts. AL, axial length; SV, single vision.

Figure 2; Table 4 demonstrate the age-specific annual axial elongation for the SV 2012_13 and 2018_19 cohorts across different percentiles. Overall, axial growth decreases with increasing age in both cohorts. The 25th, 50th, and 75th percentile curves are slightly steeper in the 2012_13 cohort, whereas the 5th and 95th percentile curves are steeper in the 2018_19 cohort, suggesting that the slowest- and fastest-growing eyes in the 2018_19 cohort exhibited greater changes in axial elongation over time (Fig. 2). At the 50th percentile, the 2018_19 cohort tends to show slightly lower axial elongation in younger children (ages 3–7) but slightly greater axial growth in older children and adolescents (ages 10–17) compared to the 2012_13 cohort–a trend similarly observed at the 25th and 75th percentiles. In both cohorts, median (50th percentile) annual axial growth was ≥ 0.40 mm at ages 3–5, ≥ 0.30 mm at ages 6–8, ≥ 0.20 mm at ages 9–10, ≥ 0.10 mm at ages 11–13, and < 0.10 mm at ages 14–17 (Table 4).

**Table 4:** Age-specific annual axial growth (mm/year) at different percentiles in the SV 2012_13 and SV 2018_19 cohorts.

Age, years	SV 2012_13 (*n* = 330)					SV 2018_19 (*n* = 165)				
	5th	25th	50th	75th	95th	5th	25th	50th	75th	95th
3	0.15	0.35	0.50	0.66	0.89	0.24	0.33	0.47	0.62	0.96
4	0.13	0.32	0.46	0.61	0.83	0.21	0.30	0.44	0.58	0.89
5	0.12	0.29	0.42	0.56	0.77	0.18	0.27	0.40	0.53	0.83
6	0.10	0.26	0.38	0.52	0.71	0.15	0.25	0.37	0.49	0.76
7	0.08	0.23	0.34	0.47	0.65	0.12	0.22	0.33	0.45	0.70
8	0.07	0.20	0.30	0.42	0.59	0.10	0.19	0.30	0.40	0.64
9	0.05	0.17	0.26	0.37	0.53	0.07	0.17	0.26	0.36	0.57
10	0.03	0.15	0.21	0.32	0.47	0.04	0.14	0.23	0.32	0.51
11	0.02	0.12	0.17	0.28	0.41	0.01	0.11	0.19	0.27	0.44
12	0.00	0.09	0.13	0.23	0.35	− 0.02	0.08	0.16	0.23	0.38
13	− 0.02	0.06	0.09	0.18	0.29	− 0.04	0.06	0.12	0.19	0.32
14	− 0.04	0.03	0.05	0.13	0.23	− 0.07	0.03	0.09	0.15	0.25
15	− 0.05	0.00	0.01	0.08	0.17	− 0.10	0.00	0.05	0.10	0.19
16	− 0.07	− 0.03	− 0.03	0.04	0.11	− 0.13	− 0.02	0.02	0.06	0.12
17	− 0.09	− 0.06	− 0.07	− 0.01	0.05	− 0.16	− 0.05	− 0.02	0.02	0.06

Values are presented at the 5th, 25th, 50th, 75th and 95th percentiles for each age, estimated using quantile regression models.

The baseline characteristics of the two cohorts stratified by age group are presented in Supplementary eTable 2. Patients aged 8–12 years in the 2012_13 cohort were significantly more myopic and had significantly longer AL and follow-up duration than those with similar age range in the 2018_19 cohort (all *P* < .05). The 2012_13 cohort also had a significantly more myopic SER in the 3–7 age group (*P* = .007).

**Fig. 3:**
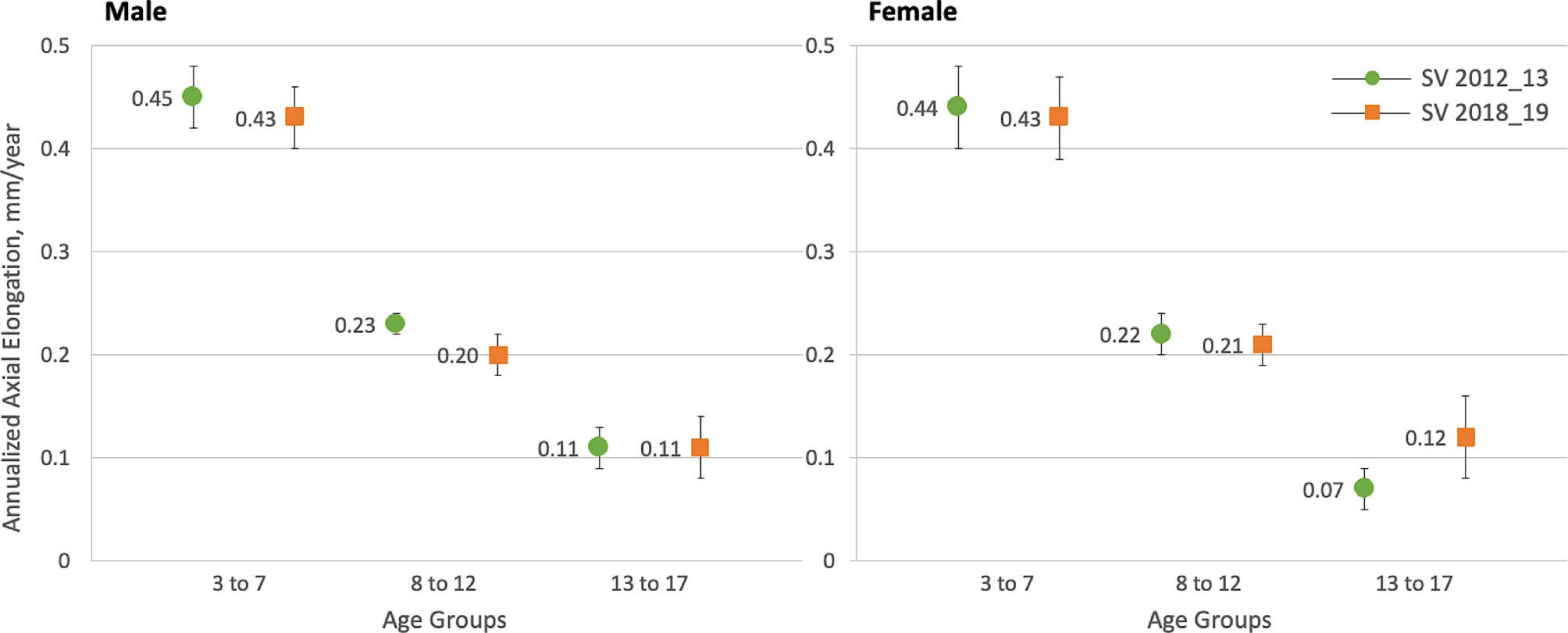
Annualized axial elongation in the SV 2012_13 and SV 2018_19 cohorts, stratified by sex and age groups. Symbols denote mean and error bars are standard error (SE).

Annualized axial elongation and myopia progression according to age group and sex are shown in Table 5; Fig. 3. Within each cohort, annualized axial growth was significantly different among the three age groups (Kruskal–Wallis test, *P* < .001). Patients aged 3–7 years had the fastest AL change (median [IQR]: 0.42 [0.30, 0.55] and 0.41 [0.30, 0.55] mm/year), followed by age groups 8–12 years (median [IQR]: 0.20 [0.11, 0.30] and 0.19 [0.11, 0.31] mm/year) and 13–17 years (median [IQR]: 0.08 [0.03, 0.14] and 0.10 [0.05, 0.13] mm/year) for the respective cohorts (Table 5).

**Table 5:** Unadjusted and adjusted annualized AL and SER changes in the SV 2012_13 and SV 2018_19 cohorts, stratified by age groups.

Age groups, years	SV 2012_13 (*n* = 330)			SV 2018_19 (*n* = 165)			*p* value (*p*^a^, *p*^b^, *p*^c^)
	3–7^a^ (*n* = 98)	8–12^b^ (*n* = 180)	13–17^c^ (*n* = 52)	3–7^a^ (*n* = 62)	8–12^b^ (*n* = 76)	13–17^c^ (*n* = 27)	
Unadjusted model, median [IQR]^φ^							
AL change, mm/year	0.42^‡^ [0.30–0.55]	0.20^†^ [0.11–0.30]	0.08^†‡^ [0.03–0.14]	0.41^‡^ [0.30–0.55]	0.19^†^ [0.11–0.31]	0.10^†‡^ [0.05–0.13]	0.980, 0.904, 0.482
SER change, D/year	− 0.67^‡^ [− 0.95 to − 0.27]	− 0.35^†^ [− 0.62 to − 0.17]	− 0.15^†‡^ [− 0.29 to 0.00]	− 0.60^‡^ [− 0.97 to − 0.28]	− 0.37^†^ [− 0.57 to − 0.18]	− 0.13^†‡^ [− 0.29 to − 0.09]	0.755, 0.960, 0.784
Adjusted model, mean (SE)^#^							
AL change, mm/year^ξ^	0.45 (0.03)	0.22 (0.01)	0.09 (0.01)	0.43 (0.02)	0.21 (0.01)	0.12 (0.02)	0.650, 0.315, 0.207
SER change, D/year^τ^	− 0.67 (0.06)	− 0.42 (0.02)	− 0.17 (0.03)	− 0.61 (0.06)	− 0.37 (0.03)	− 0.19 (0.04)	0.433, 0.151, 0.631

^φ^Mann–Whitney U test was used for between-cohort comparisons, *P < .05; Kruskal–Wallis test with bonferroni post hoc correction was used for within-cohort comparisons; ^†^significantly different from age group 3 to 7, *P* < .017; ^‡^significantly different from age group 8 to 12, *P* < .017. ^#^GEE model, *P < .05. ^ξ^Mean (SE) adjusted for age, follow-up duration and baseline AL as covariates. ^τ^Mean (SE) adjusted for age, follow-up duration and baseline SER as covariates. AL, axial length; D, dioptres; SE, standard error; SER, spherical equivalent refraction; SV, single vision.

In the 2012_13 cohort, the model-adjusted axial elongation was 0.45 (0.03) mm/year in the 3–7 age group, 0.22 (0.01) mm/year in the 8–12 age group, and 0.09 (0.01) mm/year in the 13–17 age group. Similarly, in the 2018_19 cohort, it was 0.43 (0.02), 0.21 (0.01), and 0.12 (0.02) mm/year, respectively (Table 5). Power analysis indicated that the achieved power for age subgroup comparisons exceeded 95% in the 2012_13 cohort and 90% in the 2018_19 cohort. The GEE model showed no significant group × age group interaction effect (*P* = .480), indicating that annualized axial elongation did not differ significantly between cohorts across age groups. A similar trend in axial growth was observed in both sexes, with no significant group × age group × gender interaction effect (*P* = .792), suggesting that annualized axial elongation was comparable between male and female participants across all age groups in both cohorts (Fig. 3).

In the 2018_19 cohort, 34 patients switched to DIMS spectacles after wearing SV spectacles for at least 12 months because of fast myopia progression and axial elongation. Due to the small sample size, their results are presented in the Supplementary Information (eTables 3–5, eMethods, eResults, and eDiscussion).

## Discussion

This retrospective study is among the first to present axial elongation patterns in myopic children and adolescents in Hong Kong, based on data from a clinical setting. An annual axial growth of 0.27 mm and 0.26 mm was found in the 2012_13 and 2018_19 cohorts, respectively, comprising individuals aged 3–17 years who wore SV spectacles. This aligns closely with recent studies showing a consistent annual elongation of 0.29 mm in Hong Kong children before the COVID-19 pandemic^16,17^. Additionally, our axial elongation rate was comparable to that of 0.25 mm/year observed in Chinese children with non-progressive myopia^18^. By contrast, a European study found in individuals with accelerated eye growth an annual axial elongation of 0.30 mm but in those with more consistent eye growth an elongation of 0.13 mm^19^. Hence, our finding of 0.26–0.27 mm/year axial elongation may be classified as non-progressive compared to the Chinese study but as progressive compared to the European study. This suggests a distinct eye growth pattern between Asians and Caucasians, potentially attributable to the higher prevalence of myopia in Asia (60%) than in Europe (40%)^20^.

The estimated annual axial elongation in the 2012_13 cohort was 0.45 mm for ages 3–7, 0.22 mm for ages 8–12, and 0.09 mm for ages 13–17. In the 2018_19 cohort, the corresponding values were 0.43, 0.21 and 0.12 mm, respectively. Compared with other studies, Chen et al.^21^ reported a mean axial elongation of 0.80 mm over three years (0.27 mm/year) in Singaporean children aged 3–6 years. Truckenbrod et al.^22^ documented a 0.30 mm annual axial growth in 3-year-old German children. The notably higher axial elongation found in our younger age group might be attributed to a greater proportion of progressive myopes in our sample. Using predictive models from the Orinda Longitudinal Study of Myopia (OLSM)^23^ in the United States and the Singapore Cohort Study of the Risk Factors for Myopia (SCORM)^24^, Chamberlain et al.^12^ estimated age-specific axial elongation in myopes. Their virtual cohorts aged 8–12 years exhibited annual axial elongation of 0.25 mm (OLSM) and 0.26 mm (SCORM), slightly higher than our findings for the same age group. This discrepancy may be attributed to the preference of university clinic optometrists in prescribing myopia control interventions to children in this age group, thus leaving those with relatively slower or stable myopia progression to wear SV spectacles. Consistent with our findings, previous studies reported an annual axial growth of approximately 0.10 mm in teenagers aged 13 years and older^22,25,26^.

Age at baseline was the most significant factor influencing axial elongation, regardless of sex. Both our raw data and model-based estimations consistently show that younger children experience the fastest axial growth, which progressively slows with age. Each year of increase in baseline age results in a decrease in annualized AL change by 0.04 mm (Table 3), a trend also reflected in our quantile regression model-estimated age-specific annual axial growth across different percentiles (Table 4). At the 50th percentile, the estimated annual axial elongation was 0.50 mm at age 3, gradually decreasing to − 0.07 mm at age 17 in the 2012_13 cohort, and 0.47 mm at age 3, decreasing to − 0.02 mm at age 17 in the 2018_19 cohort. A recent retrospective analysis of eight longitudinal studies in China reported that each additional year of baseline age was associated with a decrease in annualised axial elongation by approximately 0.05 to 0.06 mm. Their estimated axial growth rates for myopes range from 0.58 mm/year at age 6 to 0.36 mm/year at age 10, and further reduce to 0.09 mm/year at age 16^27^. However, these estimates were notably higher than ours, possibly due to the inclusion of six RCTs among the eight studies, in which the authors themselves reported a difference of 0.02 mm in axial growth estimates when study design (RCT vs. population-based study) was factored into their GEE model. These differences suggest that, despite all our study samples being of Chinese ethnicity, population or regional variations may also contribute to differences in axial growth rates among myopic children and adolescents. This underscores the importance of our study in capturing the axial growth patterns in Hong Kong.

In recent years, axial elongation data from control groups in myopia control trials have emerged as a potential source of reference growth data^28^. When comparing our findings with those of age-matched control groups wearing SV spectacles in RCTs^15,29–34^, the annualized axial elongation of these control groups was generally greater than ours by at least 30%. Similarly, a meta-analysis incorporating data from control groups across more than 40 RCTs estimated that the annual axial elongation among Asian children aged 8–12 years ranged from approximately 0.50–0.25 mm and decreased to approximately 0.22–0.11 mm at 13–17 years^13^. These values are also much higher than those estimated in our study, which may be attributed to the potential bias inherent in clinical trials, where participants are often recruited based on specific and stringent criteria. RCTs are an established method to accurately assess the true effect of an intervention^35^. However, given the increasing availability of proven myopia control interventions, the inclusion of untreated control groups in future myopia studies may raise ethical concerns^36^. A high dropout rate may also occur when participants are not assigned to a treatment^37^ One solution proposed by Bullimore et al.^37^ is to adopt a virtual control group. In the three-year DIMS follow-up study, Lam et al.^38^ incorporated a group of historical SV spectacle lens wearers to serve as a comparison for their treatment group. Nonetheless, the sample size for their historical control group was limited. Hence, our findings may serve as reference data for virtual control groups in future myopia control trials.

Our study found no significant differences in the axial elongation between sexes. This finding aligns with that of a previous study on emmetropes, concluding that sex is not a predictive factor for AL changes during normal eye development^39^. The Correction of Myopia Evaluation Trial (COMET)^26^ study and Li et al.^40^ also documented a similar axial growth rate between boys and girls. Additionally, there is evidence that no gender differences were found in axial elongation and myopia progression in Hong Kong children^41^. By contrast, several studies suggested that annual axial elongation is notably faster in girls^27,42,43^. Chen et al.^18^ reported that axial elongation is not influenced by sex in non-progressive myopes but is significantly faster in female progressive myopes. Therefore, the effect of sex on axial elongation remains controversial. Future research with larger sample sizes and more comprehensive data, including puberty-related factors such as height and weight, may provide further insights into this issue.

The estimated myopia increase in our two cohorts was − 0.47 and − 0.41 D/year. Donovan et al.^44^ compiled myopia progression data from 20 RCTs using cycloplegic autorefraction and found that Asian children exhibited a myopia progression of − 0.82 D/year. The large difference from our findings may be attributed to recruitment bias in myopia control trials. Moore et al.^28^ reported myopia progression rates of − 0.67 and − 0.18 D/year at ages 7 and 17 years in a clinical setting in Ireland. This deviation from our results might be due to the lack of cycloplegic refraction data in younger participants. In our study, approximately 35% of all patients underwent cycloplegic refraction during their first visit, primarily younger children. Noncycloplegic refraction tends to overestimate myopia and underestimate hyperopia compared with cycloplegic refraction, with more pronounced differences in younger children^45^. However, given the retrospective nature of our study conducted in a clinical setting, cycloplegic refraction data were not available for all participants at all follow-up visits. Consequently, this study focused on axial elongation rather than myopia progression. In a survey of UK-based optometrists, 34% agreed with performing cycloplegic refractions in children aged 2–4 years on their first visit but only 25% agreed with it in children aged 5–7 years. For this age group, 9.6% of optometrists supported cycloplegic refractions at each subsequent visit^46^.

Despite the proven efficacy of myopia control spectacles lenses and contact lenses in reducing myopic axial elongation throughout childhood^47^, eye care practitioners across the globe still frequently prescribe SV spectacles to young myopes^48^. This discrepancy in practice may be attributed to the varying levels of awareness regarding available myopia control options among practitioners and parents across countries and regions^48^. Overall, our 2012_13 and 2018_19 cohorts demonstrated similar rates of annualized axial elongation and myopia progression, indicating that the patterns of axial growth and myopia progression among myopic children and adolescents wearing SV spectacles did not significantly change before and after the introduction of DIMS spectacles into clinical practice. This could be due to the high level of awareness and education among university clinic optometrists in managing myopia progression, even when limited myopia control interventions were available. The stable and consistent axial growth patterns observed over the past 10 years suggest that our cohorts could serve as a valuable reference for future clinical trials.

Given the rapid axial elongation observed in younger children in our cohort, practitioners must exercise greater caution when prescribing SV spectacles to young children. More frequent follow-up visits (at least every 6 months) should be scheduled for children wearing SV spectacles^49^, and myopia control strategies should be promptly implemented upon detecting a rapid or abnormal increase in axial elongation. Educating parents about the importance of increasing outdoor time^50^, while reducing screen time and near work^51^ for their children, is also crucial.

The strength of our study lies in the use of axial elongation data derived from electronic clinical records in an optometry practice, offering a better representation of axial elongation patterns in routine clinical settings. Based on reported axial elongation rates of 0.37 ± 0.16 mm before 2012^52^ and 0.29 ± 0.18 mm after 2019^17^ for Hong Kong children, we sought to achieve 90% power to detect a 0.08 mm difference in axial elongation (SD = 0.17 mm) between two cohorts at a significance level of 0.05 (two-tailed). Using G*Power analysis, the final achieved power in our study was up to 98%, given the inclusion of 330 participants in the 2012_13 cohort and 165 participants in the 2018_19 cohort. This indicates that our sample size was more than adequate to draw robust conclusions regarding the comparison of axial elongation trends between the two cohorts. Our study also provided comprehensive estimates of axial elongation across different age groups, serving as a useful reference for future myopia control trials assessing treatment efficacy.

Nevertheless, this study has several limitations. First, the study sample was obtained solely from an optometry clinic on a university campus, which may not fully reflect the general population of Hong Kong. Additionally, the axial elongation rate may have been underestimated due to university clinic optometrists’ preference for prescribing myopia control treatments rather than SV spectacles to children, potentially introducing selection bias. Future research should include a more diverse sample from various clinical settings in Hong Kong to improve the generalizability of the findings. Second, we only included individuals who presented to the clinic for new prescriptions of SV spectacles, whereas those without correction or those who did not renew their prescriptions during the study period were excluded from analyses. Individuals with stable or no myopia progression were less likely to renew their prescriptions^53^. Third, due to the retrospective nature of this study, we were unable to assess the participants’ compliance with wearing the SV spectacles. Fourth, the retrospective design raises concerns about the lack of measurement standardization, the lack of consistent cycloplegic refraction data across all patients, and lack of information on parental myopia, age of myopia onset and time spent on near work and outdoor activities, all of which may be significant factors influencing axial growth and myopia progression patterns^54^. While future prospective studies with stricter control over these factors would provide a more robust benchmark, conducting such studies has become increasingly challenging, as more children are now managed with myopia control interventions rather than wearing SV spectacles alone. This is particularly difficult in East Asia, where myopia prevalence is high and myopia control measures are widely adopted. Fifth, the inclusion of two study cohorts separated by several years may introduce variability due to changes in lifestyle or environmental factors over time, such as increased screen time and changes in educational demands. Additionally, our 2018_19 cohort was affected by the COVID-19 pandemic, in which their annual follow-up visits were reduced or postponed, thus potentially introducing variability into our estimates of axial elongation^17,55^. Lastly, this study provides axial elongation data only for myopic children and adolescents, with a significant number of non-myopic patients (baseline SER > − 0.50D) excluded from both cohorts. Comparing axial elongation patterns between myopic and non-myopic children and adolescents could be an important avenue for future research.

## Conclusions

The use of SV spectacle lenses, as prescribed by optometrists in a clinical setting, resulted in an average axial elongation of 0.26–0.27 mm/year in children and adolescents. Our findings may serve as a reference for future myopia control trials to evaluate treatment efficacy. Regular follow-up visits with young children wearing SV spectacles are essential to identify rapid axial elongation, thereby facilitating the timely implementation of myopia control management.

## Methods

### Study design and population

In this retrospective study, data were collected from a clinical setting where patients typically visited for routine eye examinations. Electronic clinical records of patients prescribed SV spectacles between July 1, 2012, and August 31, 2013 (the 2012_13 cohort) and July 1, 2018, and August 31, 2019 (the 2018_19 cohort) at the Optometry Clinic of The Hong Kong Polytechnic University were reviewed. These two cohorts were included to facilitate a comparison of axial elongation before and after the availability of a proven myopia control spectacle lens. Specifically, the 2018_19 cohort covered the period after defocus incorporated multiple segments (DIMS) spectacle lenses were proven effective to reduce myopia progression in a two-year RCT^15^, whereas the 2012_13 cohort was a randomly chosen earlier cohort to match the total number of clinical records in the 2018_19 cohort. The last follow-up date was September 30, 2016, for the 2012_13 cohort and September 30, 2022, for the 2018_19 cohort, with a maximum follow-up period of 51 months. This duration covered summer breaks over four consecutive years, during which schoolchildren and teenagers usually have their spectacles updated before starting a new academic year. All participants had previously provided informed consent during their routine visits to the university optometry clinic, where they were informed that their clinical data might be used for teaching and research purposes. This study conformed to the tenets of the Declaration of Helsinki and was approved by the Institutional Review Board of The Hong Kong Polytechnic University (HSEARS20210612001).

Data including age, sex, visit dates, spectacles prescription dates, refractive error (sphere, cylinder, and axis), and AL were extracted from clinical records (all data were extracted between October 2022 and September 2023). Refractive errors were assessed by multiple registered optometrists using subjective refraction, with or without cycloplegia. The endpoint of subjective refractive was determined using the Maximum Plus to Maximum Visual Acuity (MPMVA) principle^56^. Based on the subjective refraction data, spectacle correction was prescribed to all participants. According to the general practice of the Optometry Clinic, cycloplegic refraction was performed at the discretion of the optometrists, though it is generally encouraged for children presenting to the clinic for the first time. AL was measured using either the IOLMaster (Carl Zeiss Meditec AG) or ALScan (Nidek Co., Ltd.). The first visit of the study period during which SV spectacle lenses were prescribed was regarded as the baseline visit.

The inclusion criteria were Chinese individuals aged 3–17 years with a minimum of two visits separated by at least 12 months. The exclusion criteria were as follows: (1) missing required data, (2) baseline spherical equivalent refractive error, (SER; sphere plus one half of cylinder power) >–0.50 D, (3) anisometropia > 2.00D at baseline, (4) presence of any ocular diseases that might affect refractive development, (5) presence of tropia or decompensated phoria, (6) history of ocular trauma or ocular surgery, and (7) previous or current use of any myopia control interventions.

### Statistical analysis

The primary outcome was axial elongation. Axial elongation was defined as the difference in AL between baseline and final visits, normalized for the follow-up duration, and expressed as the annualized AL change for each patient. Myopia progression, the secondary outcome, was calculated as the SER difference between baseline and final visits. It was similarly normalized and expressed as the annualized SER change.

Data was assessed for normality using the Kolmogorov–Smirnov test. Unless otherwise specified, data are presented as the median and interquartile range (IQR). Baseline characteristics of the right and left eyes are provided in Supplementary eTable 1. The correlation between right and left eyes was examined using Pearson correlation for AL and Spearman’s rank correlation for SER. AL and SER were highly correlated between both eyes at baseline in the 2012_13 cohort (AL: *r* = .966, *P* < .001; SER: r_s_ = 0.888, *P* < .001) and the 2018_19 cohort (AL: *r* = .964, *P* < .001; SER: r_s_ = 0.809, *P* < .001). Therefore, only data from the right eyes were analyzed and presented.

To compare the baseline characteristics of SV spectacle wearers between the two cohorts, the Mann–Whitney U test or unpaired t-test was used for continuous variables, whereas Pearson’s χ^2^ test was used for categorical variables. The annualized AL and SER changes were compared between the two cohorts using the Mann–Whitney U test.

To account for the effects of age and emmetropization on axial growth and refractive error development, particularly in children^57^, quantile regression models were adopted to predict age-specific annualized axial elongation and myopia progression at different percentiles (5th, 25th, 50th, 75th, and 95th ). For better comparability with myopia control treatment groups in RCTs, patients were also categorized into three age groups of 5-year intervals (3–7, 8–12, and 13–17 years). The annualized axial elongation and myopia progression across these age groups were analyzed using the Kruskal–Wallis test with Bonferroni post-hoc correction.

A generalized estimating equations (GEE) model with robust standard error (SE) was used to estimate the annualized AL and SER changes while adjusting for covariates. These covariates included demographic and baseline characteristics such as age, sex, SER, AL and follow-up duration. The significance level was set at *P* < .05. Statistical analyses were conducted using SPSS (version 29.0, IBM).

## Electronic supplementary material

Below is the link to the electronic supplementary material.

Supplementary Material 1.

## Data Availability

The datasets used and/or analysed during the current study available from the corresponding author on reasonablerequest.

## References

[1] Agyekum, S. et al. Cost-Effectiveness analysis of myopia progression interventions in children. *JAMA Netw. Open.* **6**(11), e2340986–e2340986 (2023). 37917061 10.1001/jamanetworkopen.2023.40986 PMC10623196

[2] Holden, B. A. et al. Global prevalence of myopia and high myopia and Temporal trends from 2000 through 2050. *Ophthalmology* **123**(5), 1036–1042 (2016). 26875007 10.1016/j.ophtha.2016.01.006

[3] Ding, B. Y. et al. Myopia among schoolchildren in East Asia and Singapore. *Surv. Ophthalmol.* **62**(5), 677–697 (2017). 28359704 10.1016/j.survophthal.2017.03.006

[4] Sankaridurg, P. et al. IMI impact of myopia. *Investig. Ophthalmol. Vis. Sci.* **62**(5), 2–2 (2021). 10.1167/iovs.62.5.2 PMC8083082 33909036

[5] Vitale, S., Sperduto, R. D. & Ferris, F. L. Increased prevalence of myopia in the United States between 1971–1972 and 1999–2004. *Arch. Ophthalmol.* **127**(12), 632-9 (2009). 10.1001/archophthalmol.2009.303 20008719

[6] Williams, K. M. et al. Increasing prevalence of myopia in Europe and the impact of education. *Ophthalmology* **122**(7), 1489–1497 (2015). 25983215 10.1016/j.ophtha.2015.03.018 PMC4504030

[7] Flitcroft, D. I. et al. IMI—defining and classifying myopia: A proposed set of standards for clinical and epidemiologic studies. *Investig. Ophthalmol. Vis. Sci.* **60**(3), M20–M30 (2019). 30817826 10.1167/iovs.18-25957 PMC6735818

[8] Iribarren, R. Crystalline lens and refractive development. *Prog Retin Eye Res.* **47**, 86–106 (2015). 25683786 10.1016/j.preteyeres.2015.02.002

[9] Wolffsohn, J. S. et al. IMI—clinical myopia control trials and instrumentation report. *Investig. Ophthalmol. Vis. Sci.* **60**(3), M132–M160 (2019). 30817830 10.1167/iovs.18-25955

[10] Tideman, J. W. et al. Association of axial length with risk of uncorrectable visual impairment for Europeans with myopia. *JAMA Ophthalmol.* **134**(12), 1355–1363 (2016). 27768171 10.1001/jamaophthalmol.2016.4009

[11] Haarman, A. E. G. et al. The complications of myopia: A review and meta-analysis. *Investig. Ophthalmol. Vis. Sci.* **61**(4), 49–49 (2020). 10.1167/iovs.61.4.49 PMC7401976 32347918

[12] Chamberlain, P. et al. Axial length targets for myopia control. *Ophthalmic Physiol. Opt.* **41**(3), 523–531 (2021). 33951213 10.1111/opo.12812 PMC8252804

[13] Shamp, W. et al. Influence of age and race on axial elongation in myopic children. *Investig. Ophthalmol. Vis. Sci.* **63**(7), 257 (2022). 10.1097/OPX.0000000000002176 39259699

[14] Kostis, J. B. & Dobrzynski, J. M. Limitations of randomized clinical trials. *Am. J. Cardiol.* **129**, 109–115 (2020). 32560898 10.1016/j.amjcard.2020.05.011

[15] Lam, C. S. Y. et al. Defocus incorporated multiple segments (DIMS) spectacle lenses slow myopia progression: A 2-year randomised clinical trial. *Br. J. Ophthalmol.* **104**(3), 363–368 (2020). 31142465 10.1136/bjophthalmol-2018-313739 PMC7041503

[16] Zhang, X. et al. Myopia incidence and lifestyle changes among school children during the COVID-19 pandemic: A population-based prospective study. *Br. J. Ophthalmol.* **106**(12), 1772–1778 (2022). 34340973 10.1136/bjophthalmol-2021-319307

[17] Choi, K. Y. et al. Evaluation of an optical defocus treatment for myopia progression among schoolchildren during the COVID-19 pandemic. *JAMA Netw. Open.* **5**(1), e2143781 (2022). 35029662 10.1001/jamanetworkopen.2021.43781 PMC8760616

[18] Chen, J. et al. Axial length changes in progressive and non-progressive myopic children in China. *Graefes Arch. Clin. Exp. Ophthalmol.* **261**(5), 1493–1501 (2023). 36449076 10.1007/s00417-022-05901-5 PMC10148786

[19] McCullough, S. et al. Axial growth and refractive change in white European children and young adults: predictive factors for myopia. *Sci. Rep.* **10**(1), 15189 (2020). 32938970 10.1038/s41598-020-72240-y PMC7494927

[20] Grzybowski, A. et al. A review on the epidemiology of myopia in school children worldwide. *BMC Ophthalmol.* **20**(1), 27 (2020). 31937276 10.1186/s12886-019-1220-0 PMC6961361

[21] Chen, D. Z. et al. Axial length elongation profiles from 3 to 6 years in an Asian paediatric population: the growing up in Singapore towards healthy outcomes birth cohort study (GUSTO). *Br. J. Ophthalmol.* (2023). 10.1136/bjo-2023-323906 37726156

[22] Truckenbrod, C. et al. Longitudinal analysis of axial length growth in a German cohort of healthy children and adolescents. *Ophthalmic Physiol. Opt.* **41**(3), 532–540 (2021). 33792977 10.1111/opo.12817

[23] Jones, L. A. et al. Comparison of ocular component growth curves among refractive error groups in children. *Investig. Ophthalmol. Vis. Sci.* **46**(7), 2317–2327 (2005). 15980217 10.1167/iovs.04-0945

[24] Wong, H. B. et al. Ocular component growth curves among Singaporean children with different refractive error status. *Investig. Ophthalmol. Vis. Sci.* **51**(3), 1341–1347 (2010). 19875656 10.1167/iovs.09-3431

[25] Birte, G. et al. Age-matched analysis of axial length growth in myopic children wearing defocus incorporated multiple segments spectacle lenses. *Br. J. Ophthalmol.* bjo-2023-324508 (2023). 10.1136/bjo-2023-324508 PMC11287573 38041675

[26] Hou, W. et al. Axial elongation in myopic children and its association with myopia progression in the correction of myopia evaluation trial. *Eye Contact Lens* **44**(4), 248–259 (2018). 29923883 10.1097/ICL.0000000000000505 PMC6013843

[27] Naduvilath, T. et al. Normative data for axial elongation in Asian children. *Ophthalmic Physiol. Opt.* **43**(5), 1160–1168 (2023). 37132642 10.1111/opo.13159

[28] Moore, M. et al. Myopia progression patterns among paediatric patients in a clinical setting. *Ophthalmic Physiol. Opt.* **44**(2), 258–269 (2024). 38062894 10.1111/opo.13259

[29] Bao, J. et al. Spectacle lenses with aspherical lenslets for myopia control vs Single-Vision spectacle lenses: A randomized clinical trial. *JAMA Ophthalmol.* **140**(5), 472–478 (2022). 35357402 10.1001/jamaophthalmol.2022.0401 PMC8972151

[30] Chen, H. et al. Low-intensity red-light therapy in slowing myopic progression and the rebound effect after its cessation in Chinese children: A randomized controlled trial. *Graefes Arch. Clin. Exp. Ophthalmol.* **261**(2), 575–584 (2023). 35976467 10.1007/s00417-022-05794-4

[31] Kanda, H. et al. Effect of spectacle lenses designed to reduce relative peripheral hyperopia on myopia progression in Japanese children: a 2-year multicenter randomized controlled trial. *Jpn J. Ophthalmol.* **62** (5), 537–543 (2018). 30083910 10.1007/s10384-018-0616-3

[32] Liu, X. et al. One-year myopia control efficacy of cylindrical annular refractive element spectacle lenses. *Acta Ophthalmol.* **101**(6), 651–657 (2023). 36779428 10.1111/aos.15649

[33] Rappon, J. et al. Control of myopia using diffusion optics spectacle lenses: 12-month results of a randomised controlled, efficacy and safety study (CYPRESS). *Br. J. Ophthalmol.* (2022). 10.1136/bjo-2021-321005 PMC10646852 36126105

[34] Xiong, R. et al. Sustained and rebound effect of repeated low-level red-light therapy on myopia control: A 2-year post-trial follow-up study. *Clin. Exp. Ophthalmol.* **50**(9), 1013–1024 (2022). 36054314 10.1111/ceo.14149 PMC10086781

[35] Bothwell, L. E. & Podolsky, S. H. The emergence of the randomized, controlled trial. *N Engl. J. Med.* **375**(6), 501–504 (2016). 27509097 10.1056/NEJMp1604635

[36] Jong, M. et al. IMI 2021 yearly digest. *Investig. Ophthalmol. Vis. Sci.* **62**(5), 7–7 (2021). 10.1167/iovs.62.5.7 PMC8088231 33909031

[37] Bullimore, M. A., Brennan, N. A. & Flitcroft, D. I. The future of clinical trials of myopia control. *Ophthalmic Physiol. Opt.* **43**(3), 525–533 (2023). 36897281 10.1111/opo.13120

[38] Lam, C. S. et al. Myopia control effect of defocus incorporated multiple segments (DIMS) spectacle lens in Chinese children: Results of a 3-year follow-up study. *Br. J. Ophthalmol.* **106**(8), 1110–1114 (2022). 33731364 10.1136/bjophthalmol-2020-317664 PMC9340033

[39] Fledelius, H. C., Christensen, A. S. & Fledelius, C. Juvenile eye growth, when completed? An evaluation based on IOL-Master axial length data, cross-sectional and longitudinal. *Acta Ophthalmol.* **92**(3), 259–264 (2014). 23575156 10.1111/aos.12107

[40] Li, T., Jiang, B. & Zhou, X. Axial length elongation in primary school-age children: A 3-year cohort study in Shanghai. *BMJ Open.* **9**(10), e029896 (2019). 31676647 10.1136/bmjopen-2019-029896 PMC6830838

[41] Lam, C. S. et al. A 2-year longitudinal study of myopia progression and optical component changes among Hong Kong schoolchildren. *Optom. Vis. Sci.* **76**(6), 370–380 (1999). 10416931 10.1097/00006324-199906000-00016

[42] Du, R. et al. Continued increase of axial length and its risk factors in adults with high myopia. *JAMA Ophthalmol.* **139**(10), 1096–1103 (2021). 34436537 10.1001/jamaophthalmol.2021.3303 PMC8391777

[43] Saw, S. M. et al. Eye growth changes in myopic children in Singapore. *Br. J. Ophthalmol.* **89**(11), 1489–1494 (2005). 16234459 10.1136/bjo.2005.071118 PMC1772924

[44] Donovan, L. et al. Myopia progression rates in urban children wearing single-vision spectacles. *Optom. Vis. Sci.* **89**(1), 27–32 (2012). 21983120 10.1097/OPX.0b013e3182357f79 PMC3249020

[45] Zhu, D. et al. Pre- and postcycloplegic refractions in children and adolescents. *PLOS ONE*. **11**(12), e0167628 (2016). 27907165 10.1371/journal.pone.0167628 PMC5132192

[46] Doyle, L. A., McCullough, S. J. & Saunders, K. J. Cycloplegia and spectacle prescribing in children: attitudes of UK optometrists. *Ophthalmic Physiol. Opt.* **39**(3), 148–161 (2019). 30957261 10.1111/opo.12612

[47] Sarkar, S., Khuu, S. & Kang, P. A systematic review and meta-analysis of the efficacy of different optical interventions on the control of myopia in children. *Acta Ophthalmol.* **102**(3), e229–e244 (2024). 37578349 10.1111/aos.15746

[48] Wolffsohn, J. S. et al. IMI—global trends in myopia management attitudes and strategies in clinical practice—2022 update. *Investig. Ophthalmol. Vis. Sci.* **64**(6), 6–6 (2023). 10.1167/iovs.64.6.6 PMC10155870 37126357

[49] Verkicharla, P. K. et al. The IMPACT myopia management guidelines. *Indian J. Ophthalmol.* **71**(7), 2882–2884 (2023). 37417138 10.4103/IJO.IJO_744_23 PMC10491045

[50] He, X. et al. Time outdoors in reducing myopia: A School-Based cluster randomized trial with objective monitoring of outdoor time and light intensity. *Ophthalmology* **129**(11), 1245–1254 (2022). 35779695 10.1016/j.ophtha.2022.06.024

[51] Enthoven, C. A. et al. The impact of computer use on myopia development in childhood: the generation R study. *Prev. Med.* **132**, 105988 (2020). 31954142 10.1016/j.ypmed.2020.105988

[52] Cho, P. & Cheung, S. W. Retardation of myopia in orthokeratology (ROMIO) study: A 2-year randomized clinical trial. *Investig. Ophthalmol. Vis. Sci.* **53**(11), 7077–7085 (2012). 22969068 10.1167/iovs.12-10565

[53] Alexandre, D. et al. Progression of myopia in teenagers and adults: A nationwide longitudinal study of a prevalent cohort. *Br. J. Ophthalmol.* **107**(5), 644 (2023). 34937695 10.1136/bjophthalmol-2021-319568 PMC10176358

[54] Jones, L. A. et al. Parental history of myopia, sports and outdoor activities, and future myopia. *Invest. Ophthalmol. Vis. Sci.* **48**(8), 3524–3532 (2007). 17652719 10.1167/iovs.06-1118 PMC2871403

[55] Wang, J. et al. Evaluation and Follow-up of myopia prevalence among School-Aged children subsequent to the COVID-19 home confinement in Feicheng, China. *JAMA Ophthalmol.* **141**(4), 333–340 (2023). 36821130 10.1001/jamaophthalmol.2022.6506 PMC9951104

[56] Kurtz, D. & Carlson, N. B. Refraction. in *Clinical Procedures for Ocular Examination* 111–205 (McGraw Hill-Education, New York, 2003).

[57] Wojciechowski, R. Nature and nurture: The complex genetics of myopia and refractive error. *Clin. Genet.* **79**(4), 301–320 (2011). 21155761 10.1111/j.1399-0004.2010.01592.x PMC3058260

