# Supplementary material for: Axial elongation among Hong Kong myopic children and adolescents wearing single vision spectacles from a clinical setting
Source: Sci Rep. 2025 May 3;15:15478. doi: 10.1038/s41598-025-99954-1 (PMC12049535; doi:10.1038/s41598-025-99954-1)
Supplement: Supplementary file 1 — Supplementary Material 1. [file 41598_2025_99954_MOESM1_ESM.docx]

# **Supplementary Information**

**Manuscript Title:** Axial elongation among Hong Kong myopic children and adolescents wearing single vision spectacles from a clinical setting

**Authors:** Kryshell Yu Qi Wong, Rachel Ka Man Chun and Andrew Kwok Cheung Lam

**eTable 1** Baseline characteristics of right and left eyes in the SV 2012_13 and SV 2018_19 cohorts

**eTable 2** Baseline characteristics of the SV 2012_13 and SV 2018_19 cohorts, stratified by age groups

**eTable 3** Baseline characteristics of the SV and SV-to-DIMS groups in the 2018_19 cohort

**eTable 4** Unadjusted annualized AL and SER changes in the SV and SV-to-DIMS groups of the 2018_19 cohort

**eTable 5** Adjusted annualized AL and SER changes in the SV and SV-to-DIMS (DIMS) groups of the 2018_19 cohort

**eMethods**

**eResults**

**eDiscussion**

**eReferences**

| **eTable 1** Baseline characteristics of right and left eyes in the SV 2012_13 and SV 2018_19 cohorts. | | | | | | |
| --- | --- | --- | --- | --- | --- | --- |
| **Baseline characteristics,**  **median [IQR]** | **SV 2012_13 (N = 660)** | | ***P* value** | **SV 2018_19 (N = 330)** | | ***P* value** |
|  | **Right eyes**  **(n = 330)** | **Left eyes**  **(n = 330)** |  | **Right eyes**  **(n = 165)** | **Left eyes**  **(n = 165)** |  |
| Spherical power, D^†^ | -2.00  [-3.50 to -1.00] | -1.75  [-3.25 to -1.00] | .002** | -1.25  [-2.25 to -0.50] | -1.25  [-2.25 to -0.25] | .306 |
| Cylindrical power, D^†^ | -0.75  [-1.75 to -0.25] | -1.00  [-2.00 to -0.50] | <.001** | -0.75  [-2.00 to -0.25] | -1.00  [-2.00 to -0.50] | <.001** |
| SER, D^†^ | -2.25  [-4.00 to -1.34] | -2.25  [-3.88 to -1.38] | .052 | -1.63  [-3.06 to -1.00] | -1.63  [-2.88 to -1.00] | .781 |
| Axial length, mm^‡^ | 24.38  [23.64 to 25.25] | 24.37  [23.61 to 25.26] | .241 | 24.19  [23.35 to 25.00] | 24.20  [23.42 to 24.98] | .659 |
| Abbreviations: D, diopters; IQR; interquartile range; SER, spherical equivalent refraction; SV, single vision. | | | | | | |
| Inter-eye comparisons using ^†^Wilcoxon signed-rank test or ^‡^Paired t-test, ***P*<.001. | | | | | | |

| **eTable 2** Baseline characteristics of the SV 2012_13 and SV 2018_19 cohorts, stratified by age groups. | | | | | | | |
| --- | --- | --- | --- | --- | --- | --- | --- |
| **Age group, years** | **SV 2012_13 (n = 330)** | | | **SV 2018_19 (n = 165)** | | | ***p* value**  **(*p*^a^, *p*^b^, *p*^c^)** |
|  | **3 to 7^a^**  **(n = 98)** | **8 to 12^b^**  **(n = 180)** | **13 to 17^c^**  **(n = 52)** | **3 to 7^a^**  **(n = 62)** | **8 to 12^b^**  **(n = 76)** | **13 to 17^c^**  **(n = 27)** |  |
| Age, years | 6.00^‡^  [4.00 to 7.00] | 10.00^†^  [9.00 to 11.00] | 14.00^†‡^  [13.00 to 15.00] | 6.00^‡^  [4.75 to 7.00] | 10.00^†^  [9.00 to 11.00] | 14.00^†‡^  [13.00 to 15.00] | .320, .655, .965 |
| Gender | | | | | | | |
| Male, no. (%) | 58 (59.2) | 105 (58.3) | 26 (50.0) | 36 (58.1) | 39 (51.3) | 13 (48.1) | .889, .301, .876 |
| Female, no. (%) | 40 (40.8) | 75 (41.7) | 26 (50.0) | 26 (41.9) | 37 (48.7) | 14 (51.9) |  |
| Cycloplegic refraction | | | | | | | |
| Yes, no. (%) | 28 (28.6) | 56 (31.1) | 16 (30.8) | 27 (43.5) | 25 (32.9) | 14 (51.9) | .052, .779, .067 |
| No, no. (%) | 70 (71.4) | 124 (68.9) | 36 (69.2) | 35 (56.5) | 51 (67.1) | 13 (48.1) |  |
| Spherical power, D | -1.00^‡^  [-1.56 to 0.00] | -2.25^†^  [-3.50 to -1.25] | -4.00^†‡^  [-5.19 to -2.25] | -0.50^‡^  [-1.06 to 0.25] | -1.50^†^  [-2.19 to -1.00] | -3.50^†‡^  [-5.00 to -1.50] | .019*, <.001**, .555 |
| Cylindrical power, D | -1.00^‡^  [-2.31 to -0.25] | -0.75^†^  [-1.44 to -0.25] | -1.13  [-1.50 to -0.50] | -1.75^‡^  [-2.50 to -0.50] | -0.50^†^  [-0.94 to -0.25] | -1.00^‡^  [-1.75 to -0.50] | .150, .019*, .988 |
| SER, D | -1.38^‡^  [-2.03 to -1.00] | -2.63^†^  [-4.00 to -1.63] | -4.56^†‡^  [-5.88 to -2.50] | -1.00^‡^  [-1.63 to -0.75] | -1.69^†^  [-2.63 to -1.25] | -4.25^†‡^  [-5.25 to -1.88] | .007**, <.001**, .573 |
| Axial length, mm | 23.46^‡^  [22.74 to 24.05] | 24.73^†^  [24.00 to 25.36] | 25.38^†‡^  [24.72 to 26.17] | 23.35^‡^  [22.70 to 24.17] | 24.29^†^  [23.90 to 24.99] | 25.38^†‡^  [24.41 to 25.93] | .900, .015*, .522 |
| Follow-up duration, months | 33.75  [22.00 to 40.50] | 35.00  [24.60 to 40.80] | 32.85  [24.58 to 36.65] | 27.85  [21.88 to 36.25] | 30.60  [23.93 to 36.95] | 29.00  [23.90 to 33.70] | .079, .009**, .116 |
| Abbreviations: D, diopters; IQR; interquartile range; SER, spherical equivalent refraction; SV, single vision. | | | | | | | |
| Comparison between three age groups within the same cohort by Kruskal-Wallis test with Bonferroni post hoc correction; ^†^significantly different from age group 3 to 7, *P*<.017; ^‡^significantly different from age group 8 to 12, *P*<.017. | | | | | | | |
| ^a^Comparison of age group 3 to 7 between two cohorts by Mann-Whitney U test, ***P*<.01, **P*<.05. | | | | | | | |
| ^b^Comparison of age group 8 to 12 between two cohorts by Mann-Whitney U test, ***P*<.01, **P*<.05. | | | | | | | |
| ^c^Comparison of age group 13 to 17 between two cohorts by Mann-Whitney U test, ***P*<.01, **P*<.05. | | | | | | | |

| **eTable 3** Baseline characteristics of the SV and SV-to-DIMS groups in the 2018_19 cohort. | | | | | | |
| --- | --- | --- | --- | --- | --- | --- |
| **Baseline characteristics,**  **median [IQR]** | **SV (n = 165)** | ***p^a^*** | **SV-to-DIMS (n = 34)** | | ***p^b^*** | ***p^c^*** |
|  |  |  | **SV (Pre)** | **DIMS** |  |  |
| Age, years | 9.00  [6.00 to 11.00] | .007** | 7.00  [6.00 to 8.25] | 8.00  [7.00 to 10.25] | <.001** | .965 |
| Gender | | | | | | |
| Male, no. (%) | 88 (53.30) | .441 | 15 (44.10) | | .441 | |
| Female, no. (%) | 77 (46.70) |  | 19 (55.90) | |  |  |
| Spherical power, D | -1.25  [-2.25 to -0.50] | .178 | -1.00  [-1.56 to -0.44] | -2.38  [-3.00 to -1.88] | <.001** | .001** |
| Cylindrical power, D | -0.75  [-2.00 to -0.25] | .885 | -0.88  [-1.75 to -0.25] | -1.00  [-1.88 to -0.50] | .076 | .248 |
| SER, D | -1.63  [-3.06 to -1.00] | .263 | -1.31  [-2.00 to -1.00] | -2.88  [-3.50 to -2.25] | <.001** | <.001** |
| Axial length, mm | 24.19  [23.35 to 25.00] | .025* | 23.95  [22.99 to 24.23] | 24.62  [23.74 to 24.98] | <.001** | .166 |
| Follow-up duration, months | 29.10  [23.60 to 36.10] | <.001** | 18.15  [13.48 to 21.95] | 18.70  [14.65 to 24.05] | .068 | <.001** |
| Abbreviations: D, diopters; DIMS, defocus incorporated multiple segments; IQR; interquartile range; SER, spherical equivalent refraction; SV, single vision. | | | | | | |
| Statistical significance value, ***P*<.01, **P*<.05. | | | | | | |
| *^a^*Between-group comparison between SV and SV (Pre) group by Mann-Whitney U test. | | | | | | |
| *^b^*Within-group comparison between SV (Pre) and DIMS of the SV-to-DIMS group by paired t-test or Wilcoxon Signed Ranks test. | | | | | | |
| ^c^Between-group comparison between SV and DIMS group by Mann-Whitney U test. | | | | | | |

| **eTable 4** Unadjusted annualized AL and SER changes in the SV and SV-to-DIMS groups of the 2018_19 cohort. | | | | | | |
| --- | --- | --- | --- | --- | --- | --- |
| **Outcomes** | **SV (n = 165)** | ***p*^a^** | **SV-to-DIMS (n = 34)** | | ***p^b^*** | ***p^c^*** |
|  |  |  | **SV (Pre)** | **DIMS** |  |  |
| Change in AL, mm/year | 0.25  [0.13 to 0.40] | <.001** | 0.47  [0.29 to 0.58] | 0.24  [0.09 to 0.42] | <.001** | .640 |
| Change in SER, D/year | -0.38  [-0.67 to -0.17] | <.001** | -0.78  [-1.14 to -0.64] | -0.41  [-0.75 to 0.00] | <.001** | .692 |
| Abbreviations: AL, axial length; D, diopters; DIMS, defocus incorporated multiple segments; IQR; interquartile range; SER, spherical equivalent refraction; SV, single vision. | | | | | | |
| Statistical significance value, ***P*<.01, **P*<.05. | | | | | | |
| *^a^*Between-group comparison between SV and SV (Pre) group by Mann-Whitney U test. | | | | | | |
| *^b^*Within-group comparison between SV (Pre) and DIMS of the SV-to-DIMS group by Wilcoxon Signed Ranks test. | | | | | | |
| ^c^Between-group comparison between SV and DIMS group by Mann-Whitney U test. | | | | | | |

| **eTable 5** Adjusted annualized AL and SER changes in the SV and SV-to-DIMS (DIMS) groups of the 2018_19 cohort. | | | |
| --- | --- | --- | --- |
| **Outcomes** | **SV (n = 165)** | **DIMS (n = 34)** | ***p* value** |
| Change in AL, mm/year^‡^ | 0.29 (0.01) | 0.24 (0.03) | .208 |
| Change in SER, D/year^†^ | -0.46 (0.03) | -0.39 (0.08) | .480 |
| Abbreviations: AL, axial length; D, diopters; DIMS, defocus incorporated multiple segments; SER, spherical equivalent refraction; SV, single vision. | | | |
| GEE model, *P<.05. | | | |
| ^‡^Mean (SE) adjusted for age, follow-up duration and baseline AL as covariates. | | | |
| ^†^Mean (SE) adjusted for age, follow-up duration and baseline SER as covariates. | | | |

**eMethods**

A subset of patients in the 2018_19 cohort switched to DIMS spectacles after wearing SV spectacles for at least 12 months, forming the SV-to-DIMS group. Patients with less than 6 months of DIMS follow-up were excluded from this group. Despite the small sample size, this group was included in this Supplementary Information to assess the effectiveness of DIMS spectacle lenses in a clinical setting. The annualized AL and SER changes before and after switching to DIMS were analyzed using the paired t-test or Wilcoxon’s signed-rank test. The SV-to-DIMS group was also compared with the SV group of the 2018_19 cohort using the Mann–Whitney U test. A generalized estimating equations (GEE) model with robust standard error (SE) was used to estimate the annualized AL and SER changes in the SV and SV-to-DIMS groups within the 2018_19 cohort while adjusting for covariates.

## **eResults**

In the 2018_19 cohort, 34 patients switched from SV to DIMS spectacles. These patients were compared with the SV group of the 2018_19 cohort. At baseline, patients in the SV-to-DIMS group had a slightly longer AL than those in the SV group (**Supplementary eTable 3**). The significantly faster myopia progression (median [IQR]: –0.78 [–1.14, –0.64] D/year) and axial elongation (median [IQR]: 0.47 [0.29, 0.58] mm/year; both *P*<.001) from wearing SV spectacles prompted the clinicians to prescribe DIMS to patients in the SV-to-DIMS group (**Supplementary eTable 4**). After wearing DIMS, the median annualized AL (0.47 [0.29, 0.58] vs 0.24 [0.09, 0.42] mm/year, *P*<.001) and SER (–0.78 [–1.14, –0.64] vs –0.41 [–0.75, 0.00] D, *P*<.001) changes were significantly reduced by approximately 49% and 47%, respectively. Median axial growth in DIMS wearers was now similar to the remaining SV group (median [IQR]: 0.24 [0.09, 0.42] vs 0.25 [0.13, 0.40] mm/year, *P*=.640).

After GEE model adjustments, similar annualized axial elongation and myopia progression were found in both groups (**Supplementary eTable 5**). The DIMS wearers had a mean (SE) axial elongation of 0.24 (0.03) mm/year, whereas the SV group had an axial elongation of 0.29 (0.01) mm/year (*P*=.208).

**eDiscussion**

In our SV-to-DIMS group, participants experienced an axial elongation of 0.47 mm/year when using SV spectacles. After switching to DIMS, the axial elongation rate was halved to 0.24 mm/year. Chen et al.^[1]^ suggested that an axial elongation of 0.20 mm/year has high accuracy for identifying progressive myopes. Notably, the axial growth rate in the SV-to-DIMS group was approximately 20% slower than in the SV group, though the difference did not reach statistical significance due to the limited sample size in the SV-to-DIMS group. Nevertheless, our findings are supported by Liu et al.^[2]^ concluding that although the treatment efficacy in clinical circumstances was weaker than that reported in the DIMS RCT, the reduction in myopic shift remained clinically significant. Hence, future studies with larger sample sizes are necessary to investigate the effectiveness of DIMS in retarding both axial elongation and myopia progression in clinical settings.

**eReferences**

1. Chen, J., et al., *Axial length changes in progressive and non-progressive myopic children in China.* Graefes Arch Clin Exp Ophthalmol, 2023. **261**(5): p. 1493-1501.

2. Liu, J., et al., *The Efficacy of Defocus Incorporated Multiple Segments Lenses in Slowing Myopia Progression: Results from Diverse Clinical Circumstances.* Ophthalmology, 2023. **130**(5): p. 542-550.
